# Neoadjuvant immunotherapy improves outcomes for resectable gastroesophageal junction cancer: A systematic review and meta‐analysis

**DOI:** 10.1002/cam4.7176

**Published:** 2024-05-08

**Authors:** Danzhu Wu, Liyuan Yang, Yu Yan, Zhengchen Jiang, Yinglong Liu, Peng Dong, Yajuan Lv, Siqin Zhou, Yiyang Qiu, Xinshuang Yu

**Affiliations:** ^1^ Clinical Medical College of Jining Medical University Jining Shandong Province China; ^2^ Shandong First Medical University and Shandong Academy of Medical Sciences Jinan China; ^3^ Department of General Surgery, Shandong Provincial Qianfoshan Hospital, Cheeloo College of Medicine Shandong University Jinan China; ^4^ Department of Oncology The Second Affiliated Hospital of Shandong First Medical University and Shandong Provincial Qianfoshan Hospital, Taian Jinan Shandong China; ^5^ Department of Oncology, The First Affiliated Hospital of Shandong First Medical University and Shandong Provincial Qianfoshan Hospital, Shandong Key Laboratory of Rheumatic Disease and Translational Medicine, Shandong Lung Cancer Institute National Key Laboratory of Advanced Drug Delivery and Release Systems Jinan Shandong China; ^6^ Medical College Wuhan University of Science and Technology Wuhan China; ^7^ School of Nursing, Tongji Medical College Huazhong University of Science and Technology Wuhan Hubei Province China

**Keywords:** cancer, gastroesophageal junction, meta‐analysis, neoadjuvant immunotherapy

## Abstract

**Background:**

In recent years, neoadjuvant immunotherapy (NAIT) has developed rapidly in patients with gastroesophageal junction cancer (GEJC). The suggested neoadjuvant treatment regimens for patients with GEJC may vary in light of the efficacy and safety results.

**Methods:**

A search of the Cochrane Library, PubMed, Embase, and Web of Science was completed to locate studies examining the safety and effectiveness of NAIT for resectable GEJC. We analyzed the effect sizes (ES) and 95% confidence intervals (CI) in addition to subgroups and heterogeneity. Meta‐analyses were performed using Stata BE17 software.

**Results:**

For these meta‐analyses, 753 patients were chosen from 21 studies. The effectiveness of NAIT was assessed using the pathological complete response (pCR), major pathological response (MPR), and nodal downstage to ypN0 rate. The MPR, pCR, and nodal downstage to ypN0 rate values in NAIT were noticeably higher (MPR: ES = 0.45; 95% CI: 0.36–0.54; pCR: ES = 0.26; 95% CI: 0.21–0.32; nodal downstage to ypN0 rate: ES = 0.60; 95% CI: 0.48–0.72) than those of neoadjuvant chemotherapy (nCT) or neoadjuvant chemoradiotherapy (nCRT) (MPR < 30%; pCR: ES = 3%–17%; nodal downstage to ypN0 rate: ES = 21%–29%). Safety was assessed using the treatment‐related adverse events (trAEs) incidence rate, surgical delay rate, surgical complications incidence rate, and surgical resection rate. In conclusion, the incidence of trAEs, incidence of surgical complications, and surgical delay rate had ES values of 0.66, 0.48, and 0.09, respectively. These rates were comparable to those from nCT or nCRT (95% CI: 0.60–0.70; 0.15–0.51; and 0, respectively). The reported resection rates of 85%–95% with nCT or nCRT were comparable to the mean surgical resection rate of 90%.

**Conclusion:**

NAIT is an effective treatment for resectable GEJC; additionally, the level of NAIT toxicity is acceptable. The long‐term effects of NAIT require further study.

## 1 INTRODUCTION

Gastroesophageal junction cancer (GEJC) is defined as adenocarcinoma (AC) with the epicenter within 5 cm from the GEJ. Globally, GEJC has become more common in recent years, ranking as the fifth‐most frequent cancer overall and the fourth‐most frequent cause of cancer‐related death.^1^ Obesity, gastroesophageal reflux disease, and premalignant Barrett's epithelium are thought to be related to GEJC.^2^ Surgery is still the main treatment for GEJC. Surgery alone, however, is frequently linked to high rates of metastasis and recurrence in addition to poor prognosis in individuals with locally advanced EC.^3^ Therefore, treatment for GEJC has drawn growing interest from academics. Neoadjuvant treatment can significantly lengthen patient life and lower both local and distant recurrence.^4^ Consequently, neoadjuvant treatment has been incorporated into the most recent National Comprehensive Cancer Network (NCCN) recommendations and is utilized in clinical practice.^5^ In comparison with surgery alone, a number of therapeutic methods, including perioperative chemotherapy (CT) and adjuvant or neoadjuvant chemoradiation therapy (CRT and nCRT), increase patient survival rates.^6^, ^7^ Preoperative CT was used by 14% of patients in a multicenter retrospective analysis of 315 patients with pT2‐4 EGJC, and the 5‐year OS rate was 51%.^8^ However, a more successful treatment method has not yet been found. Immune checkpoint inhibitors (ICIs) have significantly altered GEJC treatment methods during the past few years.^9^ Programmed death‐1 (PD‐1) and its ligand (PD‐L1) inhibitors, in addition to cytotoxic T‐lymphocyte antigen‐4 (CTLA‐4) inhibitors, are the main ICIs utilized in clinics. Immune T‐cell responses to malignancies can be activated by blocking these regulatory mechanisms.^10^, ^11^ Most NAIT clinical trials for resectable GEJC, including ECOG‐ACRIN and MATTERHORN, are still continuing at this time. Data from some trials have been presented at international conferences hosted by the American Society of Clinical Oncology (ASCO), the European Society of Medical Oncology (ESMO), and other organizations. According to the preliminary findings of this research, NAIT is effective and prolongs survival in patients with resectable GEJC.^12^ Consequently, a meta‐analysis of published data from clinical trials involving NAIT will offer suggestions for neoadjuvant treatment and directions for upcoming clinical trials.

This meta‐analysis attempts to demonstrate the efficacy and safety of NAIT for resectable GEJC based on the available data. The findings of this analysis offer recommendations for a thorough GEJC treatment plan.

## 2 MATERIALS AND METHODS

### 2.1 Data sources and search strategy

A computerized search on the PubMed, Cochrane, Web of Science, EMBASE, and ClinicalTrials databases was used to search articles reporting on the evaluation of the efficacy of NAIT for resectable GEJC and for the most recent data on ongoing clinical trials of NAIT for GEJC at international oncology conferences such as ASCO and ESMO. The search keywords included “Gastroesophageal Junction Cancer” and Mesh words such as “Esophagogastric Junction,” “Neoplasms,” “Neoadjuvant,” and “Immunotherapy.” By expanding the “Title/Abstract” search limit, the number of successful literature searches was boosted. To ensure completeness and prevent missing papers, references from all articles connected to this meta‐analysis were carefully reviewed by reading the title, abstract, and/or full text. Using Endnote X9 software, the articles were collated and classified by the two researchers independently. Manual reviews of references from pertinent conference reports were also conducted. The Preferred Reporting Items for Systematic Reviews and Meta‐Analyses (PRISMA) 2020 and AMSTAR‐2 criteria were followed for conducting the current meta‐analysis. The present systematic review and meta‐analysis were registered in PROSPERO (registration no. CRD42023420046).

### 2.2 Inclusion and exclusion criteria

The following represent the characteristics of the articles included in the current meta‐analysis: (I) stage I‐III resectable patients with histologically confirmed gastroesophageal conjugate carcinoma who had a distinct pathological and radiological diagnostic; (II) in clinical practice or registered clinical studies, ICIs are used; and (III) the inclusion of some or all of the primary research outcomes, including pCR, MPR, nodal downstage to ypN0 rate, an incidence of trAEs, and the rates of surgical delay, surgical resection, surgical complications, and others. The following were the exclusion criteria: (I) literature presented as cases, reviews, or editorials; (II) GEJC patients had localized tumor progression or tumor metastasis that made surgery impossible; (III) fewer than 10 participants were included in the research; (IV) the studies used repeated or overlapped data; (V) NAIT was not adequately studied for both safety and effectiveness; and (VI) the primary conclusions of the current meta‐analysis were not addressed by the studies.

### 2.3 Data abstracted

Based on the predetermined inclusion and exclusion criteria, two investigators independently reviewed the literature. Any disagreement was resolved by consultation or by a third reviewer. To assure the authenticity and integrity of the data, each article underwent several evaluations. Furthermore, a standard format for extracting the parameters was used, including first author, year of publication, ClinicalTrials.gov identifier, intervention model, study phase, ICIs, median age, gender proportionality, and sample size. The primary and secondary outcome endpoints are MPR, pCR, nodal downstage to ypN0 rate, incidence of trAEs, surgical resection rate, incidence of surgical complications, and the surgical delay rate. The parameters were reported as “NR” if information could not be obtained from these investigations.

### 2.4 Statistical analysis

The meta‐analysis was carried out using Stata BE17 software. The pCR, MPR, and nodal downstage to ypN0 rate were employed as the main impact indicators because the included studies were primarily single‐arm clinical trials. Effect measurements include the 95% CI and ES. Heterogeneity was determined using a chi‐squared (χ^2^) test and *Q*‐test (*I* ^2^). The random effect model was applied if the heterogeneity is significant; otherwise, the fixed‐effect model was utilized. Statistics were deemed significant at *p* < 0.05. The source of the heterogeneity was investigated using subgroup analysis.

### 2.5 Assessment of study quality and publication bias

Due to the fact that the bulk of the pertinent research was single‐arm clinical trials, and the included studies were evaluated for quality using the MINORS checklist, or Methodological Index for Non‐randomized research.^13^ (eTable 1 in the Supplement) utilizing the Cochrane Risk of Bias Tool for randomized controlled trials (RCT), the quality of the randomized controlled trials was assessed.^14^ (eTable 2 in the Supplement) The quality of the literature was assessed independently by two investigators. The judgment of a third researcher was considered if there was a disagreement between them.

## 3 RESULTS

### 3.1 Literature search results

**FIGURE 1:**
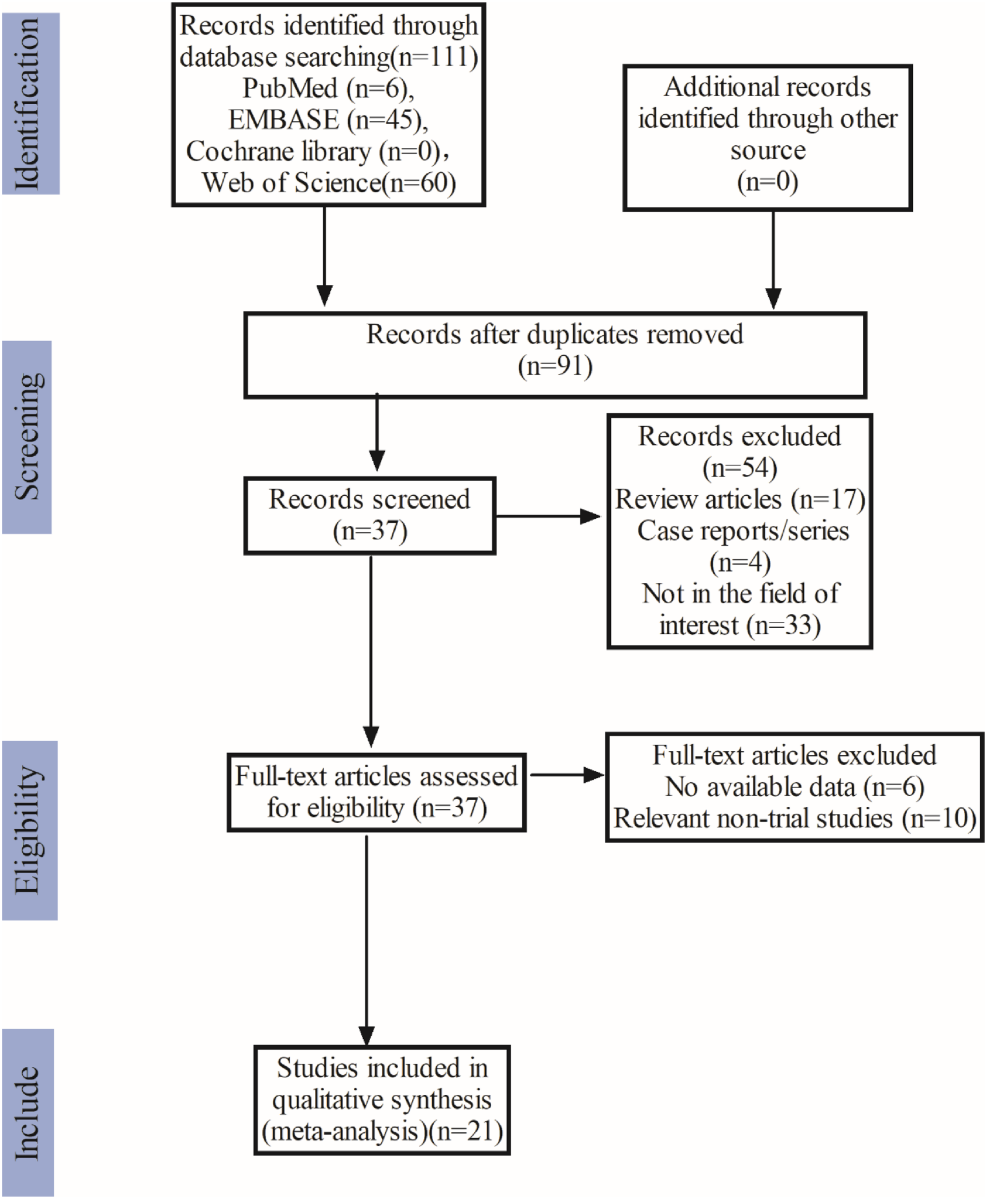
Results of the literature search.

One hundred eleven studies were selected from the initial search based on the research strategy listed in the methodology section, and 20 duplicate publications were discarded. On the basis of their titles and abstracts, 54 papers were also disqualified. Thirty‐seven papers were ultimately chosen for thorough and in‐depth analysis. Sixteen studies were then excluded after a careful reading of the entire text because they did not match the requirements for inclusion. In the end, 21 articles with 753 patients total were used for the analysis. The listed specifics of the studies are presented in Table 1. Of the 21 included studies, 16 were single‐arm cohort studies. The selection procedure for the study is depicted in Figure 1.

**TABLE 1:** Summary of the characteristics of studies on NAIT in resectable GEJC.

First author	Published year	ClinicalTrials.gov identifier	Intervention model	Study phase	ICI	Sample size	Median age	Proportion of gender	MPR	pCR	Nodal downstage to ypN0 rate
André^15^	2022	NCT04006262	Single‐arm	II	Nivolumab+ipilimumab	32	65 (40–80)	Male 23 (71.9%) Female 9 (28.1%)	72.4% (21/29)	58.6% (17/29)	48.3% (14/29)
Jiang^16^	2022	NCT04065282	Single‐arm	II	Sintilimab+chemo (paclitaxel and capecitabine)	36	65.5 (43–76)	Male 24 (66.7%) Female 12 (33.3%)	47.2% (17/36)	19.4% (7/36)	58.3% (21/36)
Tang^17^	2022	NCT03631615	Single‐arm	II	Camrelizumab+chemo (oxaliplatin and capecitabine) + RT	36	65.5 (35–72)	Male 28 (77.8%) Female 8 (22.2%)	48.5% (16/33)	36.4% (12/33)	77.8% (28/36)
Yin^18^	2022	NCT04890392	Single‐arm	II	Tislelizumab+chemo(oxaliplatin and S‐1)	32	60.5 (35–74)	Male 27 (84.4%) Female 5 (15.6%)	56.7% (17/ 30)	26.7% (8/30)	65.6% (21/32)
Zhu^19^	2022	NCT02730546	Single‐arm	Ib/2	Pembrolizumab+chemo (carboplatin and paclitaxel) + RT	31	62 (44–76)	Male 30 (96.8%) Female 1 (3.2%)	NR	24.1% (7/29)	NR
Du^20^	2022	NCT04061928	Single‐arm	II	Toripalimab+chemo+RT	24	NR	NR	72.2% (13/18)	33.3% (6/18)	NR
Ko^21^	2022	NCT03165994	Single‐arm	II	Sotigalimab+chemo (carboplatin /paclitaxel) + RT	34	NR	NR	64.3% (18/28)	35.7% (10/28)	NR
Uboha^22^	2022	NCT03490292	Single‐arm	I/II	Avelumab+chemo (carboplatin and paclitaxel) + RT	22	64	Male 20 (90.9%) Female 2 (9.1%)	42.1% (8/19)	26.3% (5/19)	NR
Wei^23^	2022	ChiCTR1900024428	Single‐arm	II	Sintilimab+chemo+RT	18	NR	NR	33.3% (6/18)	44.4% (8/18)	NR
Sun^24^	2022	NCT03488667	Single‐arm	II	Pembrolizumab+chemo (oxaliplatin、leucovorin and 5‐fluorouracil)	37	NR	Male 30 (81.1%) Female 7 (18.9%)	NR	18.5% (5/27)	NR
Tang^25^	2022	NCT04755543	Single‐arm	Ib	LP002 + chemo(cisplatin and 5‐fluorouracil)	30	64.5 (50–74)	Male 26 (86.7%) Female 4 (13.3%)	NR	NR	NR
Al‐Batran^26^	2022	NCT03421288	Two‐arm	IIb	Atezolizumab+chemo (5‐Fluorouracil, leucovorin, oxaliplatin and docetaxel)	146	NR	NR	48.6% (71/146)	24.0% (35/146)	NR
Verschoor^27^	2022	NCT03448835	Single‐arm	II	Atezolizumab+chemo (docetaxel, oxaliplatin, and capecitabine)	20	62 (47–77)	Male 18 (90.0%) Female 2 (10.0%)	70.0% (14/20)	45.0% (9/20)	NR
Liu^28^	2022	ChiCTR2000030610	RCT	II	Camrelizumab+chemo (5‐Fluorouracil, leucovorin, oxaliplatin and docetaxel)	33	63 (28–72)	Male 20 (76.9%) Female 6 (23.1%)	19.2% (5/26)	11.5% (3/26)	46.2% (12/26)
Jiang^29^	2022	NCT04119622	Single‐arm	II	Toripalimab+chemo (oxaliplatin and capecitabine)	35	61 (34–72)	Male 28 (80.0%) Female 7 (20.0%)	14.8% (4/27)	11.1% (3/27)	NR
Xueweiing^30^	2022	ChiCTR210043572	Single‐arm	II	Sintilimab+chemo (oxaliplatin and S‐1)	21	56 (31–72)	Male 10 (47.6%) Female 11 (52.4%)	38.1% (8/21)	33.3% (7/21)	NR
Raufi^31^	2022	NCT02918162	Single‐arm	II	Pembrolizumab+chemo (capecitabine and oxaliplatin)	34	65	NR	44.8% (13/29)	24.1% (7/29)	NR
Ying^32^	2021	NCT03939962	Single‐arm	II	Camrelizumab+chemo (5‐Fluorouracil, oxaliplatin and leucovorin)	49	57 (29–72)	Male 35 (71.4%) Female 14 (28.6%)	22.2% (10/45)	8.9% (4/42)	NR
Hongli^33^	2021	NCT04354662	Single‐arm	II	Toripalimab+chemo (5‐Fluorouracil, leucovorin, oxaliplatin and docetaxel)	36	60	Male 24 (66.7%) Female 12 (33.3%)	42.9% (12/28)	25.0% (7/28)	NR
Thierry Alcindor^34^	2021	NCT03288350	Single‐arm	II	Avelumab+chemo (docetaxel, cisplatin and 5‐FU/mDCF)	28	NR	Male 25 (89.3%) Female 3 (10.7%)	22.2% (6/27)	NR	NR
Li^35^	2020	NCT04341857	Single‐arm	II	Sintilimab+chemo(5‐Fluorouracil, leucovorin, oxaliplatin and docetaxel)	19	NR	NR	66.7% (6/9)	22.2% (2/9)	NR

Abbreviations: ICI, Immune checkpoint inhibitors; MPR, major pathological response; NR, not reported; pCR, pathological complete response; RCT, randomized controlled trial; RT, radiotherapy.

### 3.2 The primary outcome

#### 3.2.1 The efficacy of NAIT

**FIGURE 2:**
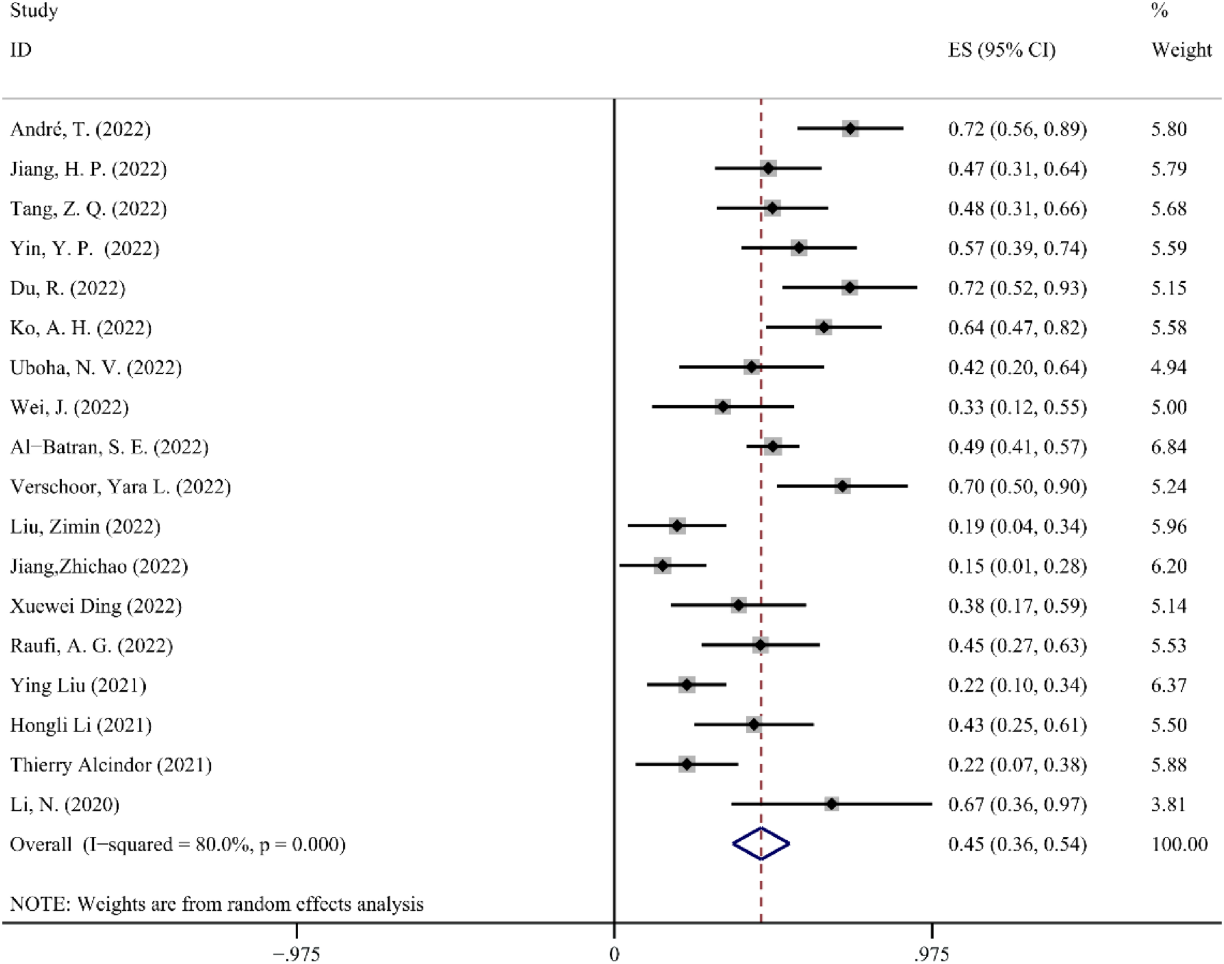
Forest map of MPR.

*MPR*. MPRs have been found in less than 10% of the primary tumors still containing live tumor cells that have been removed from patients.^36^ In the *Q*‐test, the heterogeneity result was *I* ^2^ = 80.0% (>50%) and *p* < 0.1. According to these results, this study appears to be quite heterogeneous. As a result, random effect was chosen for the meta‐analysis. NAIT appears to be effective for the MPR of resectable GEJC, and the effective rate is 0.45 according to the random effects meta‐analysis that included data from 18 studies. The effect amount summarized from these studies is 0.45 (95% CI: 0.36–0.54, *p* < 0.05). The Forest map details for MPRs are shown in Figure 2.

**FIGURE 3:**
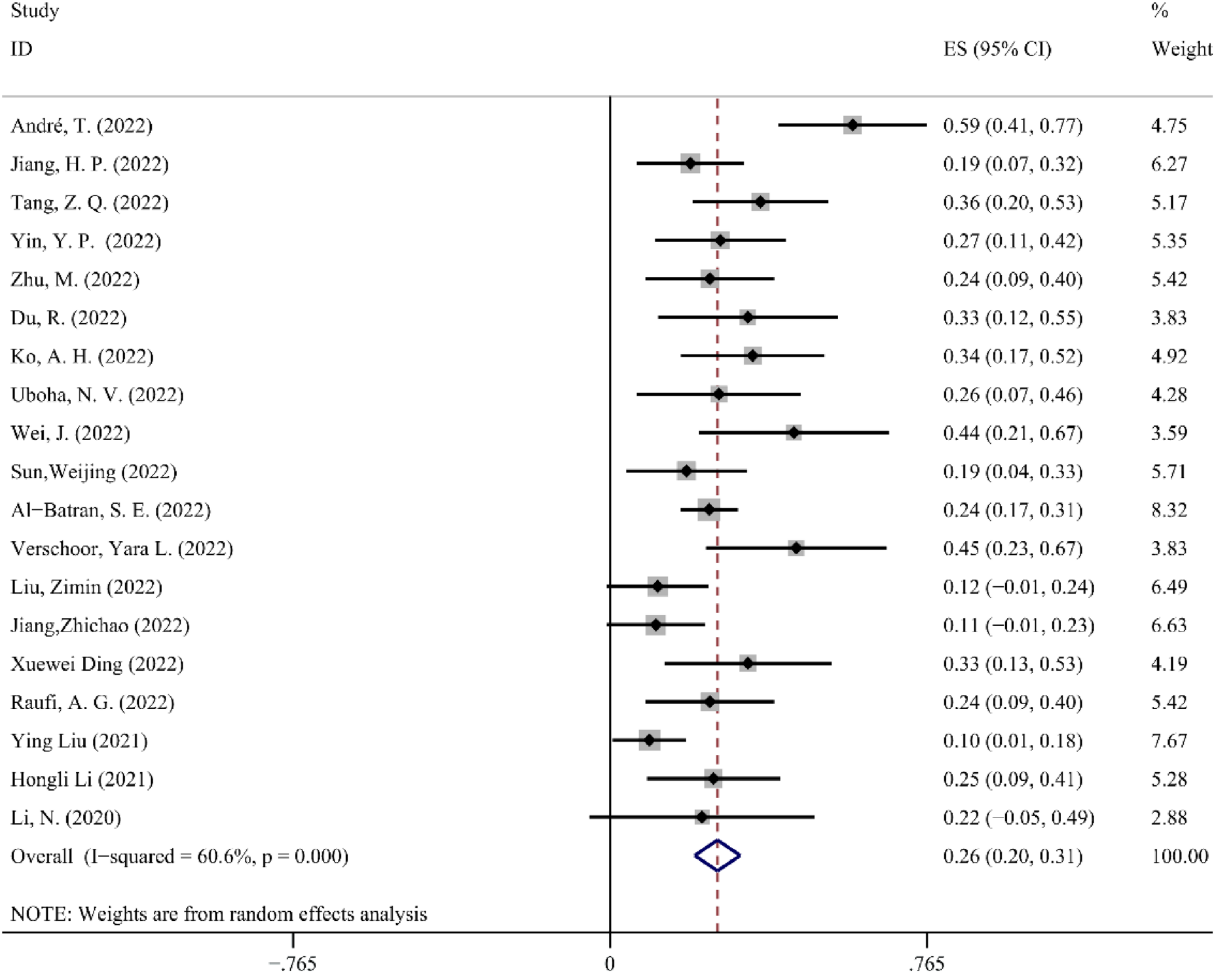
Forest map of pCR.

*pCR*. The absence of live tumor cells in the primary tumors that were surgically removed indicates pCR. pCR is another strong predictor for effectiveness of neoadjuvant therapy.^37^ Overall, the use of NAIT was supported by the combined ES of 0.26 (95% CI: 0.21–0.31) with a statistically significant change (*p* < 0.05). Because significant heterogeneity among the 19 trials (*p* < 0.1, *I* ^2^ = 60.6%) was found, a random effect model was then employed. The Forest map details for the pCR are shown in Figure 3.

**FIGURE 4:**
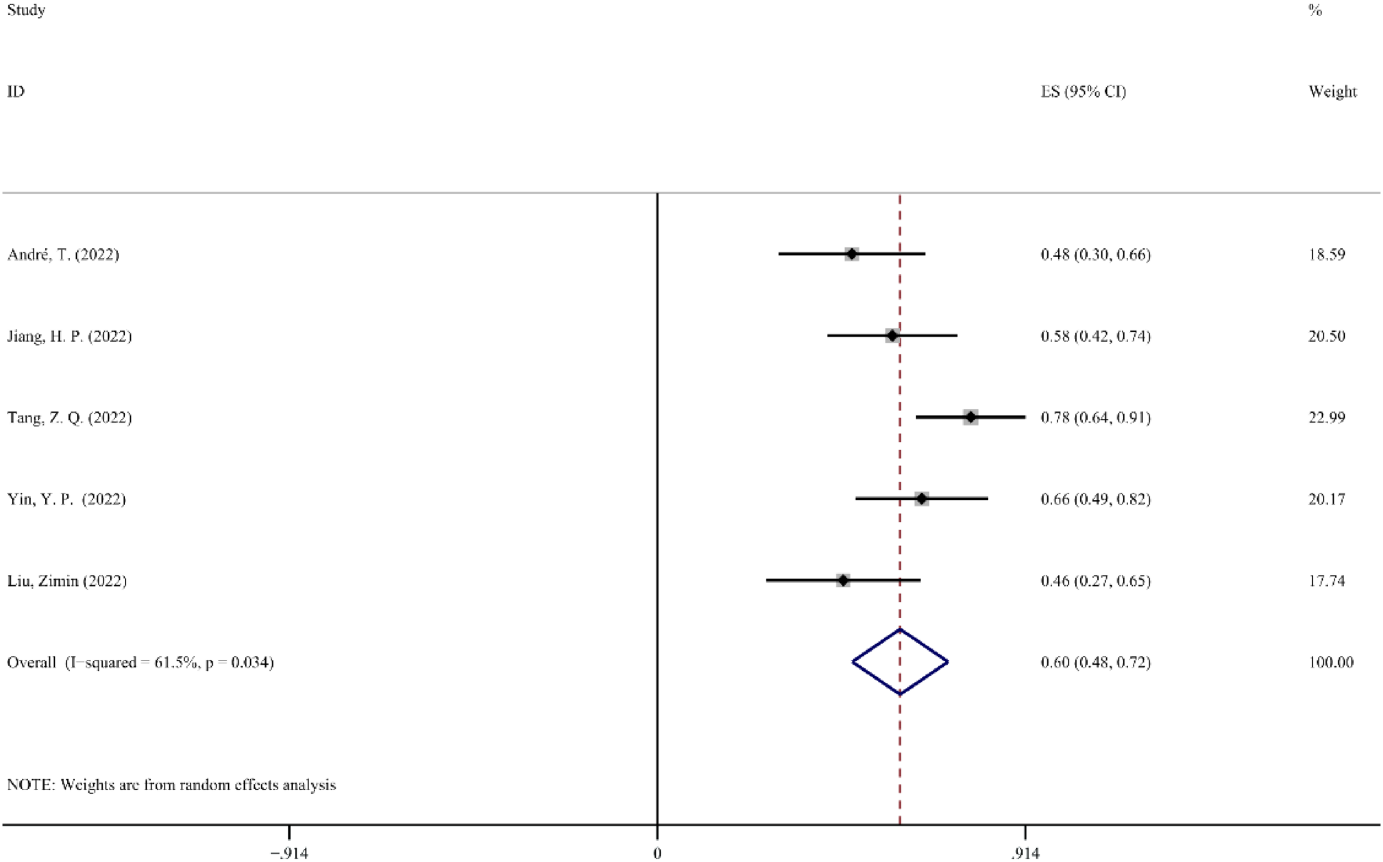
Forest map of the nodal downstage to ypN0 rate.

*Nodal downstage to ypN0 rate*. According to the report, an essential sign that preoperative therapy was successful is the nodal downstage to ypN0 rate.^38^ The nodal downstage to ypN0 rate is also an important indicator of the effectiveness of neoadjuvant therapy. The test result demonstrated significant heterogeneity (*p* < 0.1, *I* ^2^ = 61.5%); therefore, random effect was chosen for meta‐analysis. The effect amount summarized from 5 studies is 0.60 (95% CI: 0.48–0.72, *p* < 0.05). The Forest map details for the nodal downstage to ypN0 rate are shown in Figure 4.

#### 3.2.2 The safety of NAIT

**FIGURE 5:**
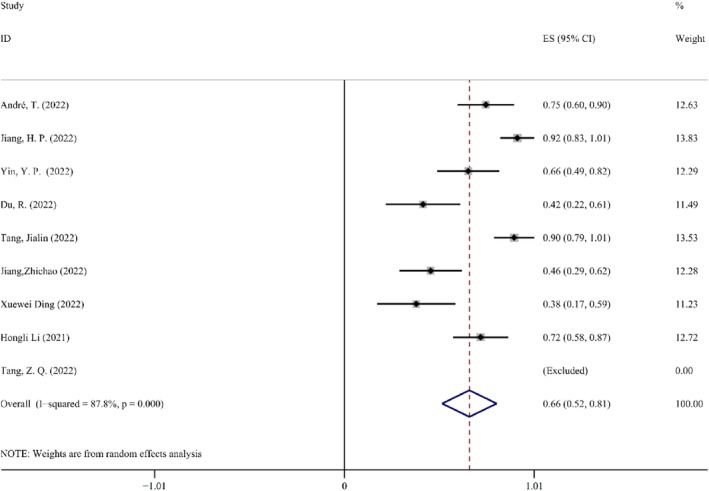
Forest map of the incidence of trAEs.

*Incidence of trAEs*. TrAEs, which reflect adverse events happening as a result of utilizing ICIs, are a crucial indicator for evaluating the safety of neoadjuvant immunotherapy. Version 4 of the National Cancer Institute Common Terminology Criteria for Adverse Events (NCICTCAE) was used to assess the incidence of trAEs. Only nine of the included 21 clinical trials provided information on the prevalence of trAEs (*N* = 282). The combined ES was 0.66 (95% CI: 0.52–0.81, *p* < 0.05). Because of the significant heterogeneity (*p* < 0.1, *I* ^2^ = 87.8%), the random effect model was used for the study. After the Xuewei Ding (2022) research was excluded, the incidence of trAEs remained unchanged, leaving an 86.4% heterogeneity. In addition, the toxicities associated with NAIT were considered manageable, with the most common trAEs being anemia, leukopenia, and neutropenia. The Forest map details for the incidence of trAEs are shown in Figure 5.

**FIGURE 6:**
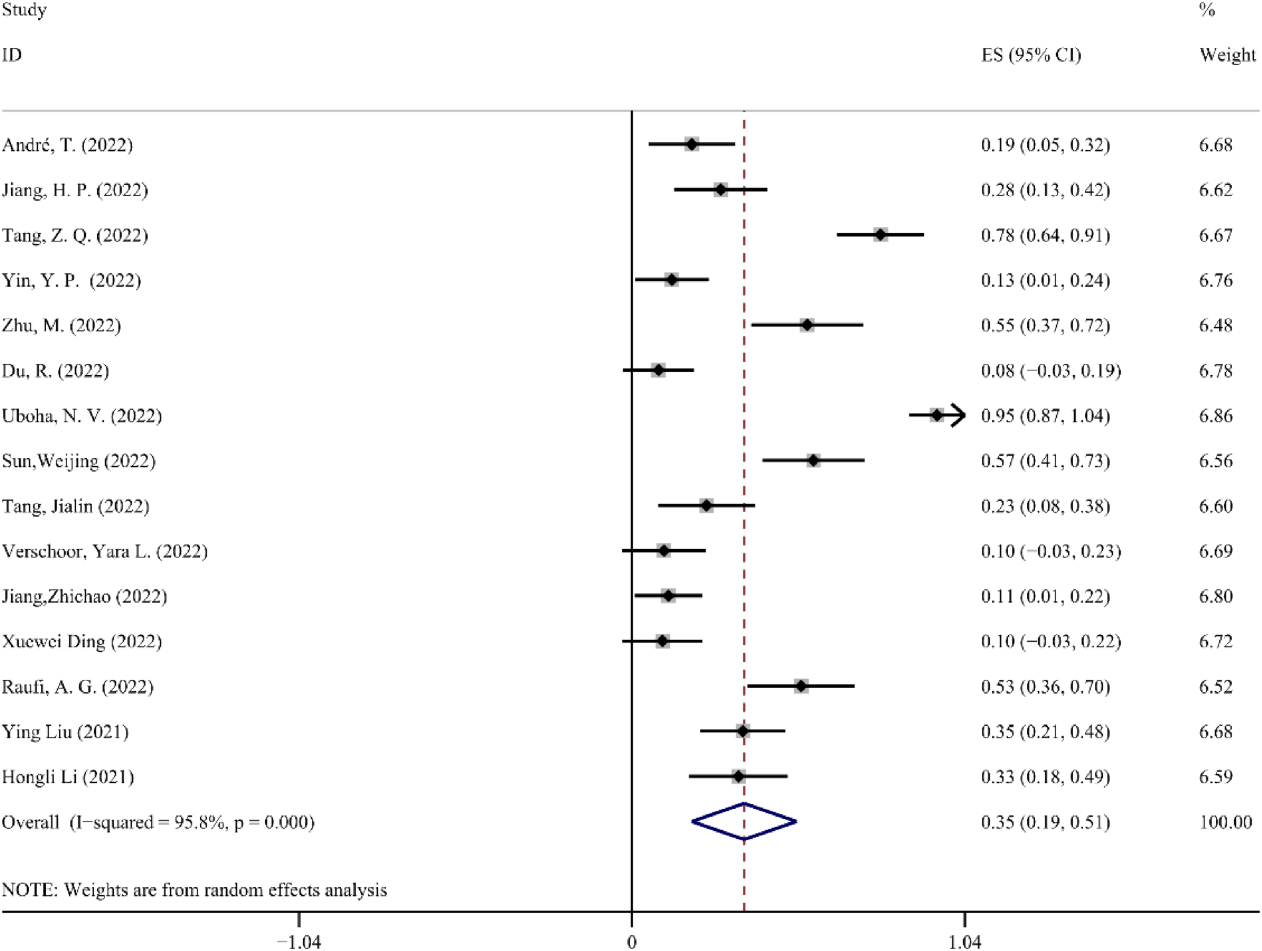
Forest map of grade 3 or higher trAEs.

*Grade 3 or Higher trAEs*. The heterogeneity test indicated *I* ^2^ = 95.8%, which was >50% with *p* < 0.1 in the *Q*‐test. After excluding the Uboha, N. V. (2022), research from the data, which had a heterogeneity of 89.9%, the findings remained unchanged. This finding indicated that the study's degree of heterogeneity was quite high. For the meta‐analysis, random effect was chosen. According to a meta‐analysis of random effects, the effect amount summarized by 15 studies was 0.35, and the 95% CI was 0.19–0.51. A patient, unfortunately, passed away 21 days after surgery from an acute response brought on by hemophagocytic syndrome and renal insufficiency.^18^ Other trAEs were generally controllable adverse events, such as anemia, leukopenia, pneumonia, and myelosuppression. These did not have significant negative effects, nor did they result in a high postoperative mortality rate. The Forest map details of Grade 3 or higher trAEs are shown in Figure 6.

**FIGURE 7:**
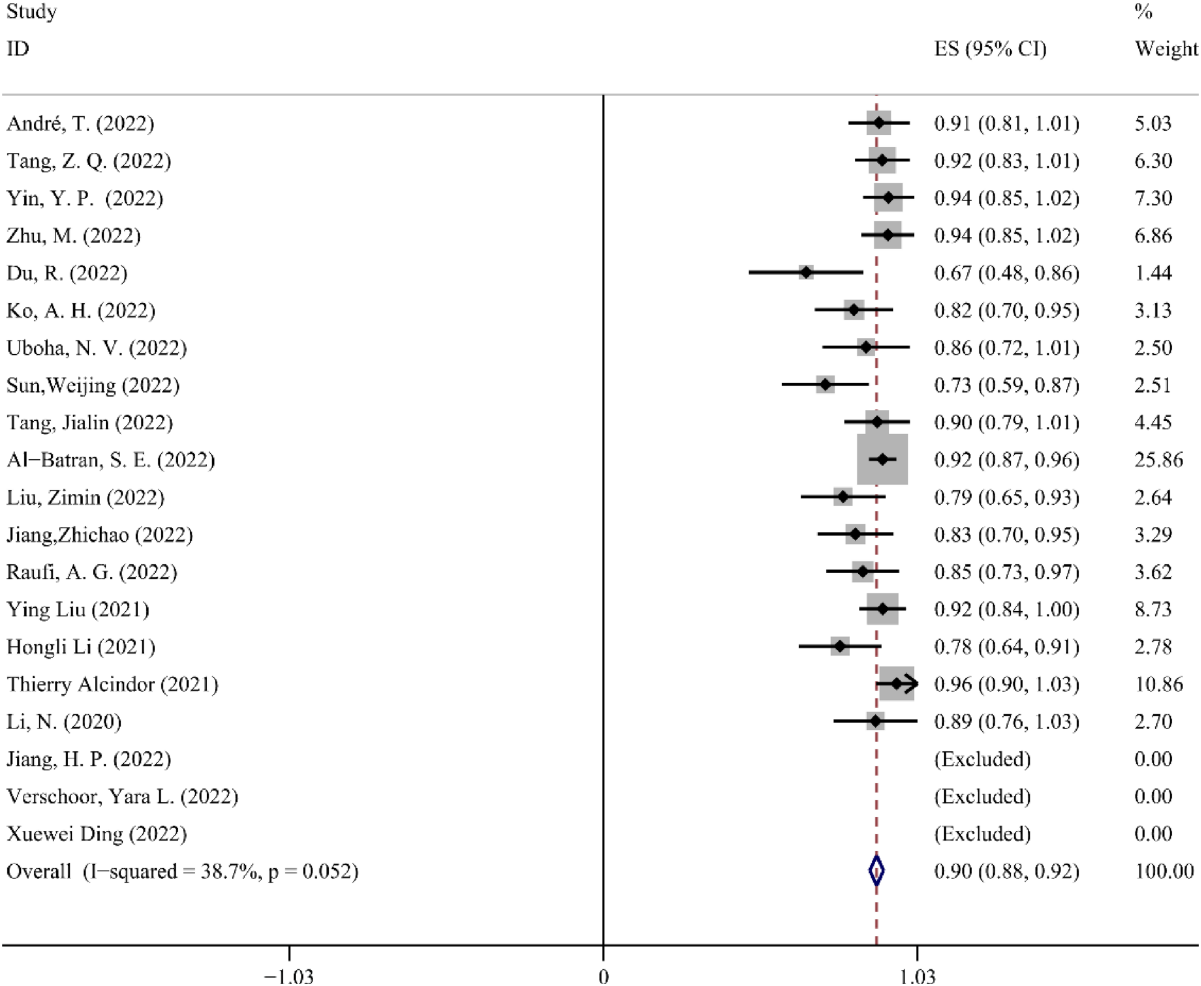
Forest map of the surgical resection rate.

*Surgical Resection Rate*. The ratio of patients who undergo surgical resection to those who are anticipated to receive surgery is known as the surgical resection rate. Another crucial metric of safety in the application of NAIT is the rate of surgical resection. The overall ES for the 20 trials that were included was 0.90 (95% CI: 0.88–0.92), with negligible heterogeneity. In order to analyze the data, the fixed‐effect model was chosen (*p* = 0.05, *I* ^2^ = 38.7%). The Forest map details of the surgical resection rate are shown in Figure 7.

**FIGURE 8:**
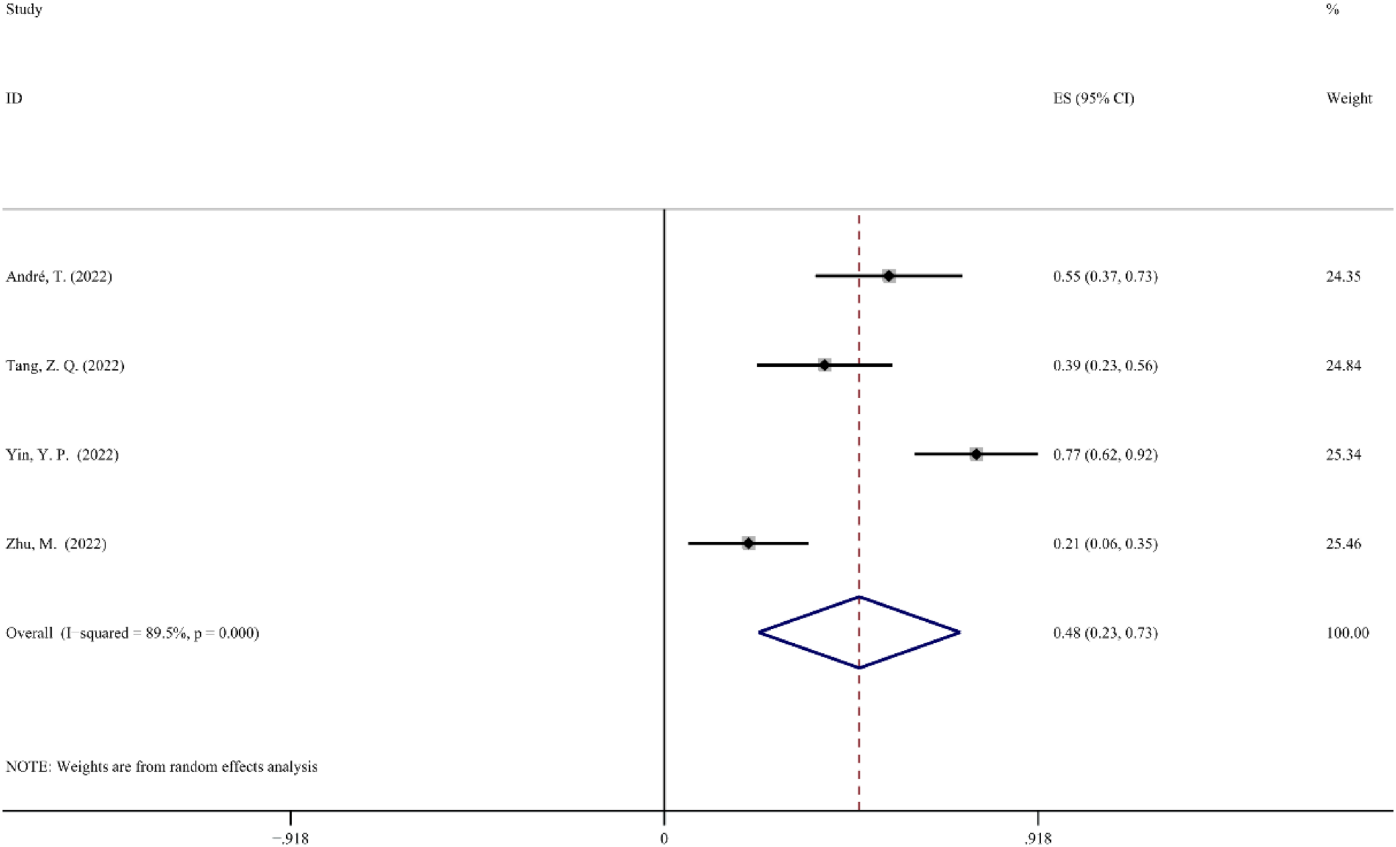
Forest map of incidence of surgical complications.

*Incidence of Surgical Complications*. To assess the safety of NAIT, consideration of the incidence of surgical complications is frequently employed, addressing postoperative complications that are connected to the surgical treatment. Four studies revealed the surgical complication rates. The random effect model was chosen for this investigation due to the heterogeneity being significantly high (*p* < 0.1, *I* ^2^ = 89.5%). The ES for these four trials was 0.48 (95% CI: 0.23–0.73). After the Yin, Y. P. (2022) research was excluded, the incidence of surgical complications remained unchanged, leaving an 76.8% heterogeneity. The most common surgical complications involved fistula, pleural effusion, and anemia in these trials. Most of these complications nonetheless had good prognoses. The Forest map details of the incidence of surgical complications are shown in Figure 8.

**FIGURE 9:**
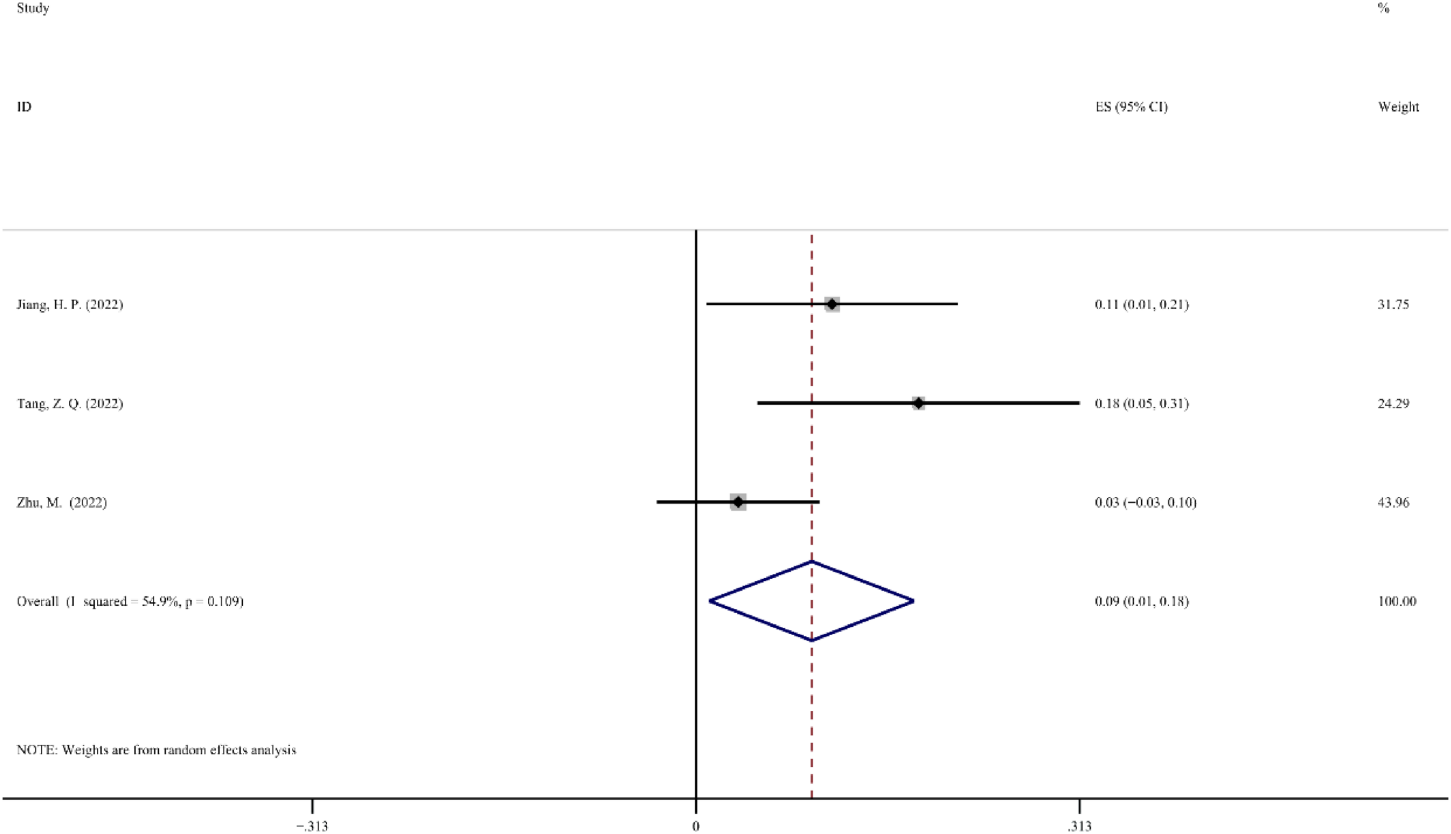
Forest map of the surgical delay rate.

*Surgical Delay Rate*. The ratio of patients who postpone surgery due to side effects from NAIT to all patients who are anticipated to undergo surgery is known as the surgery delay rate. To evaluate the safety of NAIT, the surgical delay rate is frequently used. Only three of the included five clinical trials provided information on the surgery delay rate. In these three investigations, the ES was 0.09 (95% CI: 0.01–0.18). These studies revealed remarkably heterogeneous data; hence, the random effect model was used for analysis (*p* = 0.109; *I* ^2^ = 54.9%). The Forest map details of the incidence of surgical complications are shown in Figure 9.

### 3.3 Exploratory subgroup analysis

#### 3.3.1 Subgroup analysis of the treatment mode

**FIGURE 10:**
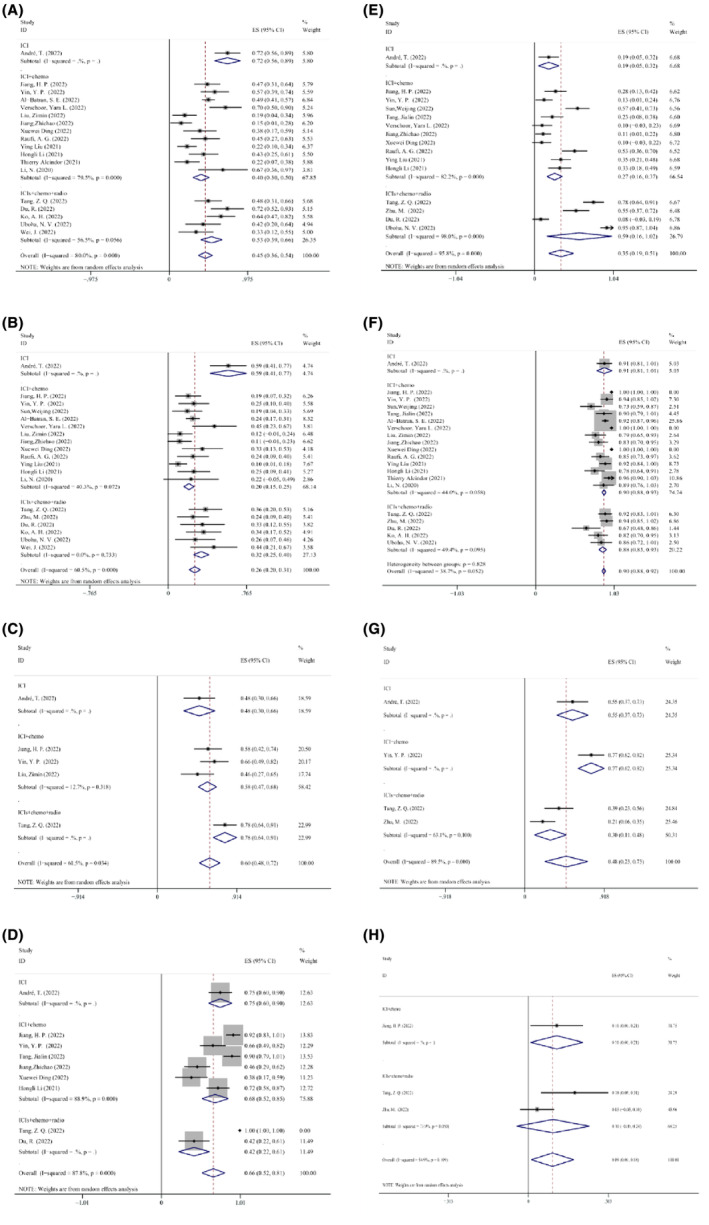
Subgroup analysis of treatment mode for (A) MPR; (B) pCR; (C) the nodal downstage to ypN0; (D) incidence of trAEs; (E) grade 3 or higher trAEs; (F) surgical resection rate; (G) incidence of surgical complications; (H) surgical delay rate.

It is important to note that only one of the 21 qualifying studies gave patients immunotherapy alone. Patients were treated in 14 of these studies using immunotherapy and chemotherapy together. Additionally, six of these trials involved the use of immunotherapy in conjunction with CRT to treat patients. Chemotherapy and CRT may change the benefits of immunotherapy; accordingly, we adopted a subgroup analysis using the treatment mode as a variable to rule out its impact on the findings of this meta‐analysis. The findings demonstrated that NAIT in conjunction with CRT was more effective in MPR and pCR and had greater safety compared with NAIT combined with chemotherapy. These findings confirmed the security and efficiency of NAIT. The Forest map details of the exploratory subgroup analysis are shown in Figure 10.

#### 3.3.2 Subgroup analysis of ICI species

**FIGURE 11:**
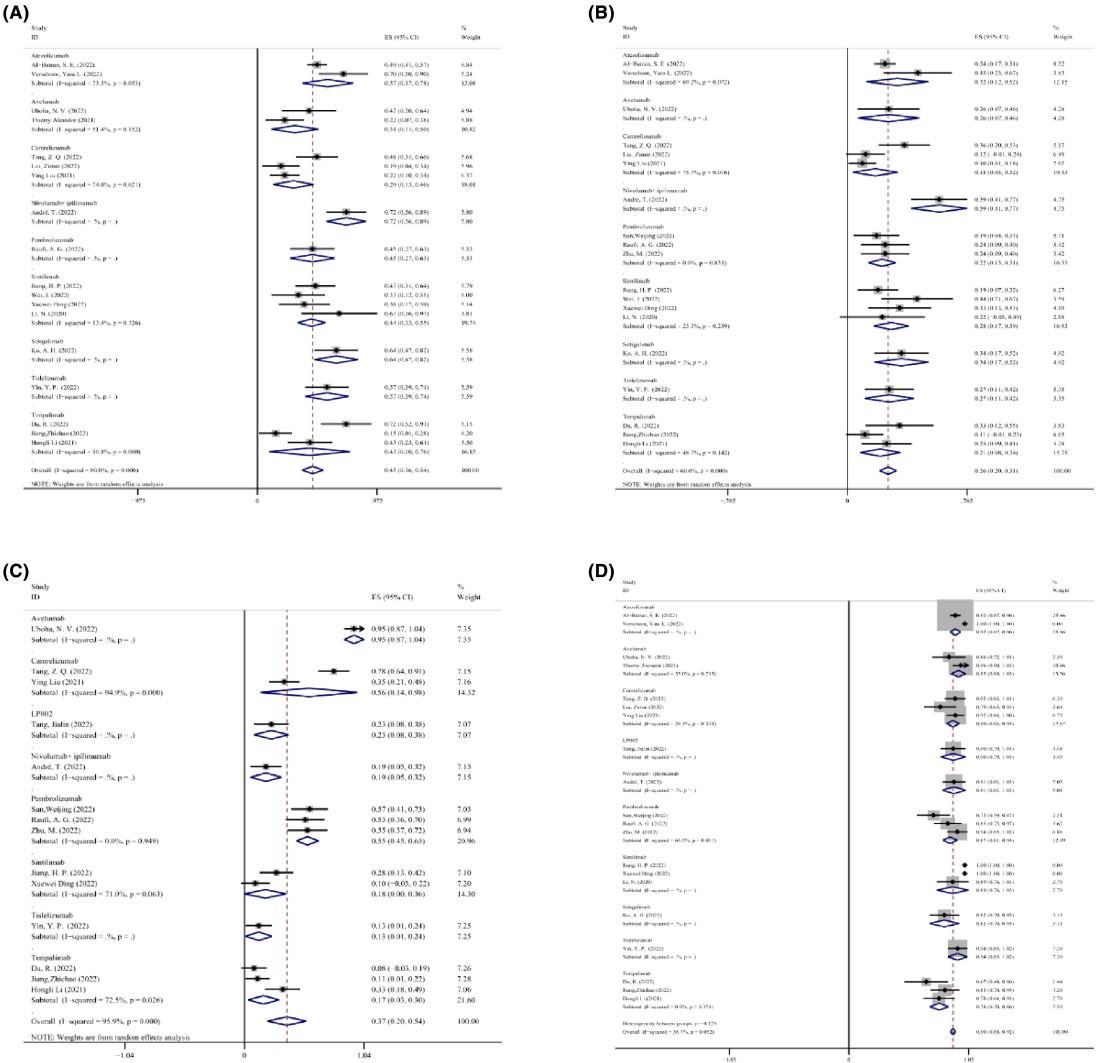
Subgroup analysis of ICI Species for (A) MPR; (B) pCR; (C) grade 3 or higher trAEs; (D) surgical resection rate.

We conducted a subgroup analysis of 21 eligible studies, including two studies where patients received atezolizumab, two studies with avelumab, three studies with camrelizumab, one study with LP002, one study with nivolumab in combination with ipilimumab, three studies with pembrolizumab, four studies with sintilimab, one study with sotigalimab, one study with tislelizumab, and three studies with toripalimab. We did so to better understand the relationship between the type of ICI and the outcome of NAIT. No statistically significant results were seen regarding the kinds of ICIs and the safety and effectiveness of NAIT, indicating that no single ICI has a clear benefit in NAIT. Forest map details of the ICI species subgroup analysis are shown in Figure 11.

## 4 DISCUSSION

Our results demonstrate that NAIT may be beneficial for GEJC patients. Extensive potential exists for improving the treatment benefits for patients with resectable GEJC when nCRT and immunotherapy are combined. To determine the potential usefulness of combined nCRT and immunotherapy in future application, we compiled the efficacy and safety data of medications from the most recent prospective trials in this systematic review and meta‐analysis.

Several solid cancers, including melanoma and non‐small cell lung cancer, have been demonstrated to respond favorably to immunotherapeutic therapies.^39^, ^40^, ^41^ The typical course of treatment for GEJC entails nCRT (carboplatin, paclitaxel, and radiation therapy) and radiation therapy, followed by surgical resection or perioperative chemotherapy (docetaxel, oxaliplatin, leucovorin, and fluorouracil; FLOT).^42^, ^43^ Unfortunately, neoadjuvant therapy has pCR rates that are often below 20%.^4^, ^44^ Based on its genomic subtype, GEJC may be amenable to immunotherapy, particularly if it is Epstein–Barr virus‐positive, MSI‐High, or chromosomally unstable.^12^, ^45^ The FDA approved pembrolizumab on September 22, 2017, at a dose of 200 mg every 3 weeks for people with advanced or metastatic GEJC.^46^ Based on the findings of the KEYNOTE‐059/Cohort 1 trial, this approval was granted.^47^ Although there is debate regarding the effectiveness of NAIT, the findings of the current trial demonstrate its effectiveness and manageable safety when used to treat resectable GEJC. NAIT has much higher efficacy than nCT or nCRT, which has an MPR of less than 30%, a pCR of between 3% and 17%,^48^, ^49^ and nodal downstage to ypN0 rate of 21%–29% in patients with resectable GEJC.^4^, ^44^, ^50^ According to our findings, NAIT toxicity is acceptable and manageable to use in resectable GEJC. In this meta‐analysis, the combined ES of the trAE occurrence risk was 66%, which was similar to the 67% toxicity of nCRT.^4^ A meta‐analysis discovered that when immunotherapy was used in neoadjuvant therapy, in contrast to NAIT alone, NAIT plus chemotherapy had a greater incidence of trAEs. Neutropenia, alopecia, and nausea were the most common all‐grade trAEs, while anemia, neutropenia, and aspartate aminotransferase (AST) increases were high‐grade trAEs; in addition, the low rates of treatment‐related surgical delays and mortality also support the benefits of NAIT.^51^ On the contrary, by combining radiotherapy with immunotherapy, radiation oncologists may take into account the viability of reducing radiation dose, restricting the target area, and including field exposures to lower the risk of fatality. Also, the average surgical resection rate was 90%, indicating that NAIT use did not lower patients' surgical resection rates relative to the 85%–95%^52^, ^53^ surgical resection rate revealed by nCT. According to the subgroup analysis of NAIT combined with nCT or nCRT, we believe NAIT in conjunction with CRT was more effective in MPR and PCR and had a greater safety compared with NAIT combined with nCT. The reason may be that radiotherapy affects the immunogenicity of the tumor microenvironment and can result in the release of tumor‐specific antigens as well as immunogenic tumor cell death.^54^ Moreover, according to the subgroup analysis of ICI species, no ICI had a significantly greater efficacy than any other. This finding requires more verification due to the limited sample size and considerable heterogeneity.

NAIT is used to treat GEJC with the primary goals of reducing tumor size and the amount of lymph nodes involved, which will limit surgical trauma, lessen tumor staging, and increase the possibility and safety of surgical resection.^55^ Neoadjuvant treatment may also decrease or eliminate cells from the tumor that are still present during surgery, which will increase patients' overall survival by reducing the likelihood of postoperative recurrence and metastasis.^56^ Neoadjuvant treatment may also decrease or eliminate cells from the tumor that are still present during surgery, which will increase patients' overall survival by reducing the likelihood of postoperative recurrence and metastasis.^57^

Before NAIT for patients with GEJC becomes the normative treatment, a number of issues need to be resolved. First, there is currently no consensus on the best course of action for NAIT in GEJC. To compare whether immunotherapy should be used alone or in conjunction with chemotherapy or radiotherapy, large randomized controlled trials (RCTs) are required. Second, given that neoadjuvant paired with adjuvant therapy has demonstrated positive clinical results and longer survival in other cancers, the viability of such combination therapy for GEJC warrants further investigation. Thirdly, it is important to compare the variations in clinical outcomes among NAIT treatments. Also, it is critical to look for biomarkers that can predict clinical results of immunotherapy for GEJC. Finally, more research is needed on the connection between pathological response and survival in GEJC.

Our meta‐analysis is subject to a number of restrictions. For instance, a few of the papers that were included provide only conference abstracts and no full texts. Also, not all of the results from the clinical trials were released, and some have not yet reached their endpoints, which could have an impact on the stability of the findings. Furthermore, considering that the majority of studies are ongoing trials, prognostic endpoints like overall survival and disease‐free survival were not addressed in this meta‐analysis. Several studies only compare NAIT with nCT, and the majority of clinical trials are single‐arm studies. Hence, RCT studies with a high sample size, across multiple centers, are required to assess these findings. Heterogeneity is a result of variances in nCT or nCRT regimens, study designs, and clinical parameter statuses.

## 5 CONCLUSION

NAIT, in combination with nCT or nCRT in patients with resectable GEJC, shows promising efficacy and controlled safety outcomes. As such, clinical evidence to support the potential widespread use of this treatment option is provided from 21 clinical trials. RCTs with long‐term follow‐up are necessary to verify the findings and advantages of NAIT, though the findings of this study can serve as a foundation for future research.

## AUTHOR CONTRIBUTIONS

**Danzhu Wu:** Conceptualization (equal); data curation (equal); formal analysis (equal); methodology (equal); software (equal); validation (equal); writing – original draft (equal); writing – review and editing (equal). **Liyuan Yang:** Data curation (equal); formal analysis (equal); writing – original draft (equal); writing – review and editing (equal). **Yu Yan:** Data curation (equal); writing – original draft (equal); writing – review and editing (equal). **Zhengchen Jiang:** Data curation (equal); writing – original draft (equal). **Yinglong Liu:** Formal analysis (equal); software (equal); writing – original draft (equal). **Peng Dong:** Software (equal); writing – original draft (equal). **Siqin Zhou:** Writing – review and editing (equal). **Yiyang Qiu:** Writing – review and editing (equal). **Xinshuang Yu:** Conceptualization (equal); funding acquisition (lead); methodology (equal); project administration (lead); supervision (lead); validation (lead); writing – review and editing (equal). **Yajuan Lv:** Conceptualization (equal); data curation (equal); writing – review and editing (equal).

## FUNDING INFORMATION

This work was supported by Clinical Research Fund of Shandong Medical Association‐Qilu Special Project (YXH2022ZX02199) and Bethune‐Cancer Radiotherapy Translational Medicine Research Fund (flzh202113).

## CONFLICT OF INTEREST STATEMENT

The authors declare that there are no conflicts of interest.

## ETHICS STATEMENT

No ethics approval was required for the current meta‐analysis.

## Supporting information


**Data S1.** Supporting Information.

**Data S2.** Supporting Information.

## Data Availability

The data that support the findings of this study are available from the corresponding author upon reasonable request.
