# Supplementary material for: Neoadjuvant immunotherapy improves outcomes for resectable gastroesophageal junction cancer: A systematic review and meta‐analysis
Source: Cancer Med. 2024 May 8;13(9):e7176. doi: 10.1002/cam4.7176 (PMC11077431; doi:10.1002/cam4.7176)
Supplement: Supplementary file 2 — Data S2. Supporting Information. [file CAM4-13-e7176-s002.docx]

# Supplement：

| **Authors** | **Q1** | **Q2** | **Q3** | **Q4** | **Q5** | **Q6** | **Q7** | **Q8** | **Q9-12** | **Score (quality)** |
| --- | --- | --- | --- | --- | --- | --- | --- | --- | --- | --- |
| André, T.2022 | 2 | 2 | 2 | 2 | 1 | 1 | 0 | 0 | / | 10 |
| Jiang, H. P.2022 | 2 | 2 | 2 | 2 | 1 | 0 | 0 | 0 | / | 9 |
| Tang, Z. Q.2022 | 2 | 2 | 2 | 2 | 1 | 2 | 0 | 0 | / | 11 |
| Yin, Y. P. 2022 | 2 | 2 | 2 | 2 | 1 | 1 | 0 | 0 | / | 10 |
| Zhu, M.2022 | 2 | 2 | 2 | 2 | 1 | 2 | 0 | 0 | / | 11 |
| Du, R.2022 | 2 | 2 | 2 | 2 | 1 | 0 | 0 | 0 | / | 9 |
| Ko, A. H.2022 | 2 | 2 | 2 | 2 | 1 | 0 | 0 | 0 | / | 9 |
| Uboha, N. V.2022 | 2 | 2 | 2 | 2 | 1 | 0 | 0 | 0 | / | 9 |
| Wei, J.2022 | 2 | 1 | 2 | 2 | 1 | 0 | 0 | 0 | / | 8 |
| Sun,Weijing 2022 | 2 | 2 | 2 | 2 | 1 | 0 | 0 | 0 | / | 9 |
| Tang, Jialin 2022 | 2 | 1 | 2 | 2 | 1 | 1 | 0 | 0 | / | 9 |
| Verschoor, Yara L.2022 | 2 | 1 | 2 | 2 | 1 | 2 | 0 | 0 | / | 10 |
| Liu, Zimin 2022 | 2 | 1 | 2 | 2 | 1 | 0 | 0 | 8 | / | 16 |
| Jiang,Zhichao 2022 | 2 | 2 | 2 | 2 | 1 | 0 | 0 | 0 | / | 9 |
| Xuewei Ding 2022 | 2 | 1 | 2 | 2 | 1 | 0 | 0 | 0 | / | 8 |
| Raufi, A. G.2022 | 2 | 1 | 2 | 2 | 1 | 2 | 0 | 0 | / | 10 |
| Ying Liu 2021 | 2 | 1 | 2 | 2 | 1 | 0 | 0 | 0 | / | 8 |
| Hongli Li 2021 | 2 | 2 | 2 | 2 | 1 | 0 | 0 | 0 | / | 9 |
| Thierry Alcindor 2021 | 2 | 1 | 2 | 2 | 1 | 1 | 0 | 0 | / | 9 |
| Li, N.2020 | 2 | 1 | 2 | 2 | 1 | 0 | 0 | 0 | / | 8 |

**eTable 1：Assessment of the quality of included studies according to MINORS.**

Quality of non-comparative studies was determined based on the first eight items; the last four items were only used to assess comparative studies.

Checklist items: 1, a stated aim of the study; 2, inclusion of consecutive patients; 3, prospective collection of data; 4, endpoints appropriate to study aim; 5, unbiased assessment of study endpoint; 6, follow-up period appropriate to the major endpoint; 7, <5% lost to follow-up; 8, adequate control group; 9, contemporary groups; 10, baseline equivalence of groups; 11, prospective calculation of study size; 12, adequate statistical analyses. Items are scored as 0 (not reported); 1 (reported but inadequate); or 2 (reported and adequate). The maximum possible score is 24 points.For non-comparative studies, an overall score > 12 = high; 8–12 = intermediate; < 8 = low. For comparative studies, > 18 = high; 12–18 = intermediate; < 12 = low.

**eTable 2：Assessment of the quality of randomized controlled trials according to Cochrane Risk of Bias Tool for Randomized Controlled Trials.**

| **Authors** | **^1^Randomization process** | **^2^Identification and recruitment** | **^3^Intervention deviation** | **^4^Missing outcome data** | **^5^Outcome measurement** | **^6^Reported results selection** | **Score (quality)** |
| --- | --- | --- | --- | --- | --- | --- | --- |
| Al-Batran, S. E.2022 | low risk of bias | low risk of bias | low risk of bias | Some concern | low risk of bias | Some concern | **Some concern** |

These domains were rated as “high risk of bias”, “low risk of bias”, or “some concerns”. Finally, an overall risk of bias was determined. The overall risk of bias was “high risk of bias” if at least one domain was deemed “high risk of bias” or if there were “some concerns” in three or more domains. The overall risk of bias was “some concerns” if there was “some concerns” in at least one domain. The overall risk of bias was “low risk of bias” if all domains were rated as “low risk of bias”.
